# Interactions between Flow Oscillations and Biochemical Parameters in the Cerebrospinal Fluid

**DOI:** 10.3389/fnagi.2016.00154

**Published:** 2016-06-29

**Authors:** Vincent Puy, Jadwiga Zmudka-Attier, Cyrille Capel, Roger Bouzerar, Jean-Marie Serot, Anne-Marie Bourgeois, Jérome Ausseil, Olivier Balédent

**Affiliations:** ^1^Biochemistry Unit, CBH, Amiens University Medical CenterAmiens, France; ^2^INSERM U1088, Research GroupAmiens, France; ^3^BioFlowImage Research Group, Jules Verne University of PicardyAmiens, France; ^4^Geriatric Unit, General HospitalSaint Quentin, France; ^5^Neurosurgery Unit, Amiens University Medical CenterAmiens, France; ^6^Medical Imaging Unit, Amiens University Medical CenterAmiens, France

**Keywords:** cerebrospinal fluid, biochemistry, phase-contrast magnetic resonance imaging, hydrocephalus, lumbar and ventricular total protein levels

## Abstract

The equilibrium between the ventricular and lumbar cerebrospinal fluid (CSF) compartments may be disturbed (in terms of flow and biochemistry) in patients with chronic hydrocephalus (CH). Using flow magnetic resonance imaging (MRI) and CSF assays, we sought to determine whether changes in CSF were associated with biochemical alterations. Nine elderly patients with CH underwent phase-contrast MRI. An index of CSF dynamics (I_dyn_) was defined as the product of the lumbar and ventricular CSF flows. During surgery, samples of CSF were collected from the lumbar and ventricular compartments and assayed for chloride, glucose and total protein. The lumbar/ventricular (L/V) ratio was calculated for each analyte. The ratio between measured and expected levels (I_bioch_) was calculated for each analyte and compared with I_dyn_. I_dyn_ varied from 0 to 100.10^3^μl^2^.s^2^. In contrast to the L/V ratios for chloride and glucose, the L/V ratio for total protein varied markedly from one patient to another (mean ± standard deviation (SD): 2.63 ± 1.24). The I_bioch_ for total protein was strongly correlated with the corresponding I_dyn_ (Spearman’s R: 0.98; *p* < 5 × 10^−5^).We observed correlated alterations in CSF flow and biochemical parameters in patients with CH. Our findings also highlight the value of dynamic flow analysis in the interpretation of data on CSF biochemistry.

## Introduction

In 1964, Dichiro (1964) described the active circulation of cerebrospinal fluid (CSF) for the first time. Over the last two decades, our understanding of CSF dynamics has been considerably improved by the use of phase-contrast magnetic resonance imaging (PCMRI). This technique enables the reliable, non-invasive, rapid measurement of CSF and blood flows in various compartments (Barkhof et al., 1994; McCauley et al., 1995; Hoppe et al., 1998; Knobloch et al., 2014).

Chronic hydrocephalus (CH) is characterized by marked ventriculomegaly and alterations in CSF flow. The incidence of CH increases with age, and the diagnosis of this condition is based on both clinical and radiological data. Magnetic resonance imaging (MRI) can reveal severe ventricular enlargement (i.e., greater than the enlargement due to brain atrophy alone) and may also evidence CSF “flow void” in the aqueduct (Bradley et al., 1996) when very high CSF velocities are caused by active hydrocephalus. Recently, new biophysical approaches have been used to characterize the role of CSF dynamics in brain function. During the cardiac cycle (CC), the CSF oscillates between the intracranial compartment and spinal canal; this compensates for the vascular changes in brain volume and thus avoids a marked increase in intracranial pressure (ICP; Bateman, 2000; Bouzerar et al., 2012; Schmid Daners et al., 2012). In healthy individuals, the oscillations between the cranium and the spinal canal involve just 10% or so of the total CSF volume (Balédent et al., 2001). In CH, the movement of CSF between ventricular, intracranial subarachnoidal and lumbar compartments may become disorganized, and patients with CH may display abnormally low or abnormally high levels of CSF pulsatility (Chaarani et al., 2013). We reasoned that the application of PCMRI may improve the diagnosis and management of patients with CH. It has been estimated that CH accounts for up to 10% of cases of dementia. Hence, CH constitutes a modifiable risk factor because it can be treated by ventriculoperitoneal shunting. The use of PCMRI enables the volume of CSF flowing through the aqueduct in either direction during the CC (i.e., the stroke volume, SV) to be measured easily. In some patients with CH, it is not clear whether ventricular dilation is due to atrophy or another active mechanism; in this particular case, some researchers consider that a high aqueductal CSF SV in patients with dilated ventricles is a good prognostic marker for the placement of a shunt (Bradley et al., 1996; Luetmer et al., 2002; Balédent et al., 2004; Scollato et al., 2008; El Sankari et al., 2011). However, other recent studies suggest that the aqueductal SV is correlated with the aqueduct area and the ventricular volume but not with the severity or duration of clinical symptoms (Ringstad et al., 2015a). The significance of the aqueductal SV is therefore still subject to debate (Bradley, 2015; Ringstad et al., 2015a,b).

In other patients with CH, there is a blockage between the ventricles and the subarachnoid spaces. This blockage is sometimes difficult to detect on conventional, morphological images. In such cases, PCMRI shows the abnormal absence of CSF oscillations in the aqueduct and thus prompt the neurosurgeon to recommend endoscopic third ventriculostomy rather than shunt placement (Stoquart-El Sankari et al., 2009).

We hypothesized that the noted hydrodynamic alterations might also alter the CSF levels of routinely measured clinical biochemical parameters. In patients with CH, PCMRI studies have shown that the CSF flows in the ventricles or/and spinal canal are modified. The putative correlations between CSF biochemistry and CSF flow dynamics have not previously been investigated. Indeed, very few publications have even looked at biochemical differences between CSF samples in the ventricular and lumbar compartments.

The blood plasma is the source of 80% of all CSF proteins (350–500 mg/l). The total protein concentration is 2.5 times higher in the lumbar CSF than the ventricular CSF because of the gradual influx of proteins moving from the choroid plexus to the lumbar spinal canal (Regeniter et al., 2009). However, the concentration of proteins synthesized in the brain is relatively uniform in all CSF compartments but can sometimes even be lower in the lumbar region than in the ventricular region (as seen with tau protein, Reiber, 1994).

In 1958, Fishman et al. showed that the CSF protein concentration was higher in the lumbar sac than in the ventricles of patients with CH. Furthermore, the protein concentration varied greatly from one subject to another. The researchers concluded that the protein concentration gradient depended (at least in part) on the relatively high permeability to albumin of the blood-CSF barrier in the spinal subarachnoid space. However, this gradient also depends on several other factors, including: (i) mixing within the ventricles and the subarachnoid space; and (ii) removal mechanisms, which may operate in some compartments but not others (Fishman et al., 1958). Similarly, Weisner and Bernhardt’s (1978) study of samples from healthy controls showed that the CSF albumin concentration was 2.2 times greater in the lumbar region than in the ventricles.

As suggested by Milhorat (1975), the ventricles’ highly complex anatomy and the equally complex system of CSF circulation within the brain may contribute to the maintenance of a balance between the various compartments (in terms of both flow rates and CSF biochemistry). We speculated that this balance might be disturbed in patients with CH and that this disturbance might (at least in part) be attributed to abnormal CSF hydrodynamics. Hence, we used flow MRI and standard CSF assays to establish whether altered CSF flow dynamics in CH were related to the CSF’s biochemical profile.

## Materials and Methods

### The Study Population

All procedures involving human subjects were performed in accordance with the 1983 and 2008 revisions of the Declaration of Helsinki. The study was conducted at Amiens University Hospital (Amiens, France). All the patients included in the present research project were informed by a medical doctor of the aim of this research. They were all free to reject their participation without any implication on the following of their care. All have signed the consent before to be included in the research population. This study was approved by the local investigational review board (CPP Nord-Ouest II, Amiens, France; reference: 2010/17). For obvious ethical reasons, it was impossible to obtain samples of ventricular and lumbar CSF from healthy volunteers; we therefore studied nine elderly, shunted patients (5 women and 4 men; mean ± standard deviation (SD) age: 73 ± 8; age range: 56–83) with a documented clinical history of CH and easy access to the CSF during the tap test and the surgical shunt procedure.

### Acquisition of PCMRI Data

**Figure 1:**
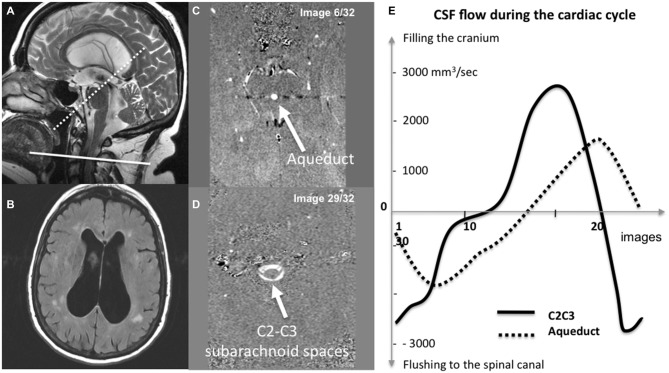
**Quantification of cerebrospinal fluid (CSF) dynamics.** A sagittal T2 weighted image **(A)** was used to select the phase-contrast magnetic resonance imaging (PCMRI) acquisition planes in patients with chronic hydrocephalus (CH) and ventricular dilation on the axial T2-weighted FLAIR image **(B)**. Images **(C,D)** respectively show CSF flowing through the cerebral aqueduct and through the cervical (C2-C3) subarachnoid spaces during the cardiac cycle (CC). Post-processing of these PCMRI data enables the CSF oscillations during the CC to be quantified **(D)**. Integration of these curves over time yields the CSF stroke volume (SV) for the aqueduct (SV_aqu_) and for the spinal canal (SV_spine_). The CSF SV corresponds to the volume of CSF moving through the slice over the CC and is the primary descriptor of CSF dynamics **(E)**.

All nine patients underwent morphological and CSF flow MRI of the brain (Table 1). All acquisitions were performed prospectively with a 3 Tesla MRI system (Signa, General Electric Medical Systems, Milwaukee, WI, USA). Conventional anatomic sequences were acquired for each patient. A sagittal T2-weighted image was used to select two PCMRI flow acquisitions (one perpendicular to the presumed direction of CSF flow through the C2-C3 subarachnoid space and the other through the cerebral aqueduct; Figure 1). Retrospective peripheral gating was used to measure 32 different time frames over the entire CC for each of the two flow acquisitions. The main PCMRI parameters were as follows: echo time: 6–9 ms; repetition time: 20 ms; flip angle: 20°; field-of-view: 16 × 12 mm^2^; pixel resolution: 0.6 × 0.6 mm^2^; slice thickness: 5 mm; views per segment: 2; velocity (encoding) sensitization: 5, 10 or 20 cm/s for the cervical aqueduct and 5 cm/s for the C2-C3 subarachnoid space. The acquisition time for each flow series was about 2 min, depending on the participant’s heart rate.

**Table 1:** **CSF flow assessment (PC-MRI) and CSF protein assay data**.

	SV_aqu_ μL/CC	SV_spine_ μL/CC	CCT_aqu_ s	CCT_spine_ s	I_dyn_ 10^3^.μL^2^.s^2^	PL g/L	PV g/L	V_exp_ g/L	PL/PV	I_bioch_ %
P1	0	630	1	1	0	0.67	0.12	0.34	5.6	−64
P2	160	650	0.7	0.7	53	0.38	0.25	0.19	1.5	31
P3	40	250	0.9	0.9	8	1.30	0.3	0.65	4.3	−54
P4	50	290	0.8	0.8	9	0.54	0.18	0.27	3	−33
P5	44	640	0.8	0.8	17	0.83	0.36	0.42	2.3	−13
P6	510	130	0.7	0.7	35	0.41	0.22	0.21	1.9	7
P7	350	430	0.8	0.8	100	0.4	0.33	0.2	1.2	65
P8	120	440	0.8	0.8	31	0.34	0.14	0.17	2.4	−18
P9	293	534	0.6	0.6	57	0.29	0.2	0.15	5	38

*Abbreviations: SV*_aqu_, *stroke volume at the aqueduct; SV*_spine_, *stroke volume through the spinal canal at C2-C3; CCT*_aqu_, *cardiac cycle time measured at the cerebral aqueduct; CCT*_spine_, *cardiac cycle time measured at the spinal canal at C2-C3; I*_dyn_,* SV*_aqu_ × *SV*_spine_ × *CCT*_aqu_ × *CCT*_spine_; *PL, lumbar CSF protein level; PV, ventricular CSF protein level; V*_exp_, *expected ventricular protein level; I*_bioch_, (*V*_m_−*V*_exp_)/*V*_exp_ *× 100*.

### Analysis of PCMRI Data

Data were analyzed using dedicated PCMRI image processing software (Balédent et al., 2001) with an optimized CSF flow segmentation algorithm. The software automatically extracts the anatomic regions of interest and calculates the corresponding flow curves over the 32 CC segments (Figure 1).

The CSF flow curves were integrated to provide the CSF SVs, which correspond to the CSF volumes displaced in both directions through the cerebral aqueduct (SV_aqu_) and the spinal canal (SV_spine_) over the CC (Nitz et al., 1992; Enzmann and Pelc, 1993). SVs are quoted in μL/CC.

An overall index of CSF dynamics (“I_dyn_”) was derived as a guide to the CSF volume displaced in the ventricles and the spinal canal during the CC. It was defined as the product of the two CSF SVs and the two CC times (CCT_aqu_ and CCT_spine_); I_dyn_ = SV_aqu_ × SV_spine_ × CCT_aqu_ × CCT_spine_.

The CCT was measured using a plethysmographic sensor worn on the patient’s finger during the MRI. The sensor monitored the changes in blood volume in the finger during the CC. The PCMRI acquisition was synchronized with the systolic pulse.

### CSF Sampling and Biochemical Analysis

All patients underwent a CSF tap test during placement of their shunt. During surgery, CSF samples (at least one for the ventricular compartment and one for the lumbar compartment) were collected from each patient. The samples were collected between 9.00 and 10.30 am after overnight fasting, in accordance with our university medical center’s validated, aseptic protocols. The plastic vials were immediately sent to the university medical center’s central laboratory. Chloride, glucose and total protein levels were determined using conventional techniques (Advia 2400 analyzer, Siemens Healthcare Diagnostics, Tarrytown, NY, USA). Briefly, chloride was assayed potentiometrically using an ion-selective electrode. The glucose level was determined in an enzymatic assay based on hexokinase and glucose-6-phosphate. The CSF total protein level was determined using pyrogallol red/acidic molybdate reagent. Levels of the resulting blue pyrogallol/molybdate/protein complex were quantified by reading the optical density at 596/694 nm. Ventricular and lumbar levels were measured for each CSF component. Under physiological conditions, the ventricular CSF protein level is around half the lumbar level (Laterre et al., 2008). We therefore defined a biochemical index (“I_bioch_”), in order to assess the difference between expected and measured protein levels in the ventricle:

(1) $$I_{\text{bioch}}=\left(V_{\text{m}}-V_{\text{exp}}\right)/V_{\text{exp}}\times 100$$

where V_m_ and V_exp_ are respectively the measured and expected ventricular CSF protein levels. V_exp_ was calculated as half the lumbar CSF protein level (based on the normal L/V ratio in healthy individuals; Laterre et al., 2008). An I_bioch_ of ~0 indicates that CSF protein is distributed normally between the ventricular and lumbar compartments, an I_bioch_ above 0 reveals a higher-than-expected ventricular CSF protein level and an I_bioch_ below 0 reveals a lower-than-expected ventricular CSF protein level.

### Statistical Analysis

A nonparametric Spearman’s test was used to assess the correlations between PCMRI and clinical biochemical parameters. A *p*-value below 0.05 was considered to be statistically significant. We also applied linear regression analysis, with extraction of the linear equation and calculation of the regression coefficient R^2^.

## Results

### PC-MRI Analysis

In the group of CH patients, PCMRI analysis revealed a wide range of CSF flow profiles (from stenosis to hyperdynamic flow) when measured at the cerebral aqueduct or at the spinal level. The mean ± SD (range) values of SV_aqu_ and SV_spine_ were 174 ± 174 (0–510) and 444 ± 188 (130–650) μL/CC, respectively. The mean values of CCT_aqu_ and CCT_spine_ were 0.8 ± 0.12 and 0.79 ± 0.12 s, respectively. The I_dyn_ values ranged from 0 to 100.10^3^ μl^2^.s^2^ (Table 1). Hence, patients with CH displayed major hydrodynamic alterations in ventricular and/or lumbar CSF compartments.

### Biochemical Variations in the Lumbar and Ventricular Compartments and their Correlation with CSF Flows

**Figure 2:**
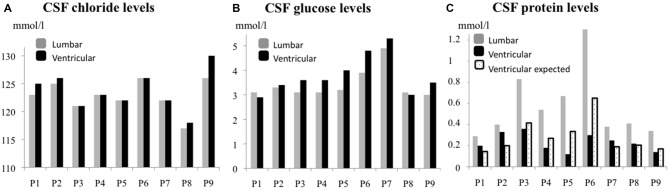
**Biochemical analyses of lumbar and ventricular CSF samples. (A)** Lumbar and ventricular CSF chloride levels in individual patients; L/V ratio ≃ 1 (mean ± standard deviation (SD): 0.99 ± 0.01). **(B)** Lumbar and ventricular CSF glucose levels in individual patients; note the absence of a significant difference between lumbar and ventricular values, L/V ratio ≃ 1 (mean ± SD mean: 0.91 ± 0.1). **(C)** Lumbar and ventricular CSF protein values in individual patients, showing a significant difference in CSF protein levels between the lumbar and ventricular compartments. The L/V ratio ranged from 1.21 to 5.58 (mean ± SD: 2.63 ± 1.46). Abbreviations: L/V, lumbar/ventricular; SD, standard deviation; [ ], concentration.

We first compared the biochemical CSF parameters in the ventricular compartment with those in the lumbar compartment (Figure 2). The mean ± SD (range) ventricular and lumbar CSF chloride levels were respectively 124 ± 3.5 (121–130) and 123 ± 2.8 (117–126) m.mol.l^−1^, the mean ventricular and lumbar CSF glucose levels were respectively 3.8 ± 0.8 (2.9–5.3) mmol.l^−1^ and 3.4 ± 0.6 (3–4.9) mmol.l^−1^, and the mean ventricular and lumbar CSF protein levels were respectively 0.23 ± 0.08 (0.12–0.36) and 0.57 ± 0.3 (0.29–1.3) mmol.l^−1^ (Table 1).

**Figure 3:**
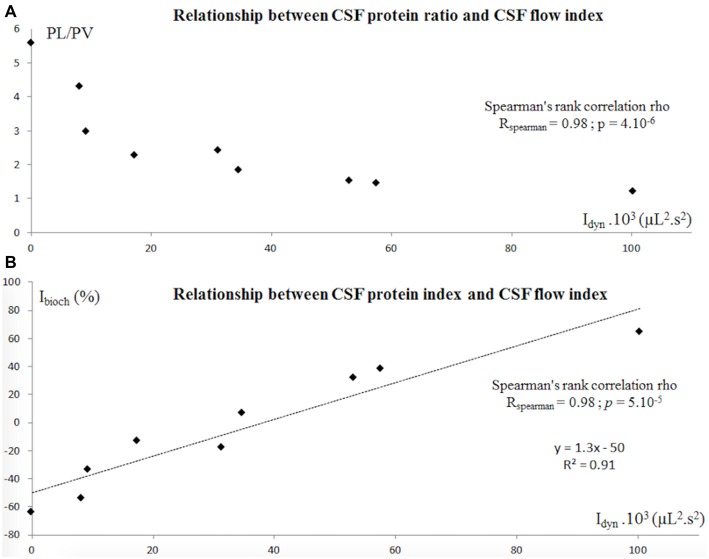
**(A)** In patients with CH, the CSF protein ratio (PL/PV) calculated for the lumbar spaces (PL) and the ventricle spaces (PV) was strongly correlated with the CSF flow from the aqueduct and the spinal spaces, represented by I_dyn_ = SV_aqu_ × SV_spine_ × CCT_aqu_ × CCT_spine_. **(B)** Similarly, I_bioch_ and I_dyn_ were strongly correlated_._ I_bioch_ = (V_m_−V_exp_)/V_exp_ × 100. An I_bioch_ of ~0 shows that CSF protein is distributed evenly between the ventricular and lumbar compartments. An I_bioch_ above 0 reveals a higher-than-expected ventricular CSF protein level, and an I_bioch_ below 0 reveals a lower-than-expected ventricular CSF protein level. Abbreviations: CH, chronic hydrocephalus; PL, lumbar protein level; PV, ventricular protein level; SV_aqu_, stroke volume measured at the cerebral aqueduct; SV_spine_, stroke volume measured at the spinal canal at C2-C3; CCT_aqu_, cardiac cycle time measured at the cerebral aqueduct; CCT_spine_, cardiac cycle time measured at the spinal canal at C2-C3; V_exp_, expected ventricular CSF protein level; V_m_, measured ventricular CSF protein level.

There were no significant ventricular vs. lumbar differences in the chloride and glucose levels. The L/V ratios for chloride and glucose were respectively 0.99 ± 0.01 and 0.91 ± 0.10. The value of the L/V protein ratio varied greatly from one patient to another but was correlated with the CSF dynamic index (Figure 3A; Spearman’s R: 0.98; *p* = 4.10^−6^). The mean value was 2.63 ± 1.24 (Figure 2). The corresponding I_bioch_ values were also widely distributed (range: −64.2% to 65%; mean ± SD: −4.5 ± 43%). Nevertheless, the respective values of I_dyn_ and I_bioch_ were highly correlated (Spearman’s R: 0.98; *p* = 5.10^−5^), suggesting the presence of a strong, linear relationship between CSF dynamics and CSF total protein levels.

## Discussion

CSF pressure and volume have long been studied in animal models of hydrocephalus and in the context of shunt design (Chahlavi et al., 2001; Pickard et al., 2005). Consequently, there are large bodies of data on pressure-volume relationships in the ventricular-subarachnoid system and the impact of CSF turnover on brain metabolism (Silverberg et al., 2010).

In animal models, it is increasingly clear that low CSF turnover impairs brain metabolism and fluid balance both early and late in life (Silverberg et al., 2001, 2003; Owen-Lynch et al., 2003; Johanson et al., 2004; Praticò et al., 2004; Nagra et al., 2008). Although a large number of studies have focused on CSF formation and turnover, the dynamics of CSF within the cerebrospinal system have not been extensively characterized (Oresković and Klarica, 2010; Pollay, 2010). Experiments in small animal models (such as mice) are complicated by the low total volume of CSF. Hence, the composition and dynamics of the CSF in the various compartments are not easy to study.

We hypothesized that CSF dynamics have an impact on CSF biochemistry. To the best of our knowledge, the present study is the first to have evaluated the association between CSF dynamics and CSF biochemistry in humans.

The present PCMRI study was designed to obtain an overview of CSF dynamics in patients with CH. Dynamic gadoteridol-enhanced MRI studies of the guinea pig have shown that: (i) movement of the tracer between the subarachnoid space and the ventricles was proportional to the CSF pressure; and (ii) that CSF turnover may be proportional to the ICP (Yamada et al., 2005). It has long been known that an increase in the mean CSF pressure is also associated with an increase in the amplitude of CSF pressure oscillations (Nornes et al., 1977; Czosnyka et al., 1988). However, the relationship between CSF pressure and CSF SV remains unclear. In patients with CH, some researchers have found that the ICP pulse wave is positively correlated with the volume of CSF movement through the cerebral aqueduct (Hamilton et al., 2012), whereas others have not observed this relationship (Ringstad et al., 2015a). The aqueductal SV accounts for only a part of the craniospinal system. Under physiological conditions, CSF oscillations through the foramen magnum influence the ICP. We consider that CSF dynamics should be studied in the subarachnoidal spaces as well as in the aqueduct. The fact that the spinal canal receives all the CSF from the ventricles and the intracranial subarachnoïd compartments (Balédent et al., 2001) prompted us to define I_dyn_, a quantitative, dynamic marker that takes account of both spinal and ventricular CSF SVs and the CC duration. In previous work, we had found that the mean ± SD CSF oscillation in a population of elderly, healthy volunteers was 34 ± 16 μL/CC in the aqueduct of Sylvius and 457 ± 147 μL/CC in the cervical spaces (Stoquart-El Sankari et al., 2007). In the current study, our PCMRI data revealed a broad range of alterations in CSF flow—ranging from abnormally slow to abnormally high. Indeed, flow at the aqueduct was either stenosed, normal or elevated (i.e., huge volume oscillations through an enlarged aqueduct; Table 1). Our data also showed that movement of the CSF in both the ventricular and lumbar compartments varies over the course of the CC.

Few studies have analyzed CSF biochemistry in both the ventricular and lumbar compartments. We showed that ventricular and lumbar CSF levels of small molecules (chloride and glucose) were similar. Indeed, electrolytes diffuse passively from the CSF to the interstitial tissue. The glucose concentration appears to be much the same through the whole cerebrospinal system. In contrast, total protein levels differed markedly when comparing the ventricular and lumbar regions. In the lumbar compartment, most CSF proteins are derived from plasma proteins (as a result of close interaction with blood vessels; Laterre et al., 2008). This mechanism might explain why protein levels are higher in the lumbar compartment than in the ventricular compartment. Distribution of various substances along the CSF spaces also depends on the rate of their removal into microvessels: faster removal corresponds to more limited distribution. When interpreting experimental results, it is important to distinguish between the movement of CSF/water (99% of the CSF’s volume is water) on one hand and the distribution of substances inside the CSF system on the other (Vladić et al., 2000; Bulat and Klarica, 2011). The flow oscillations induced by the CC cause CSF with a high protein concentration (in the lumbar compartment) to mix with CSF with a lower protein concentration (in the ventricular compartment). Given that we were unable (for ethical reasons) to measure ventricular CSF protein levels in a control group, we calculated I_bioch_ from the expected and measured ventricular protein levels. According to Laterre et al.’s (2008) study in adults, V_exp_ is half the lumbar CSF protein level under physiological conditions. I_bioch_ ranged from −64.2 to 65%, which strongly suggests that the L/V ratio varies. Interestingly, we showed that CSF protein variations were strongly correlated with the CSF flow oscillations during the CC. Our I_bioch_-I_dyn_ correlation was independent of the exact value of the physiological L/V protein ratio and thus evidenced a relationship between oscillatory movement of the CSF and variations in CSF biochemistry. The observed variations in the CSF flow patterns (I_dyn_), the L/V protein ratio and I_bioch_ strongly support this hypothesis. (Figure 3).

Lastly, the stable isotope labeling approach recently described by Lehmann et al. (2015) could be of great value for quantifying: (i) the rates of synthesis and clearance of a large range of proteins in the lumbar and ventricular CSF; and (ii) the large interindividual differences in protein concentration found in patients with CH.

In conclusion, the results of our pilot study in a reliable human model suggest that the amplitude of the CSF flow during the CC influences CSF protein levels and the distribution of protein within the central nervous system’s cavities. This proof-of-concept study opens up new avenues in the investigation of the relationship between clinical biochemical parameters and dynamic MRI parameters, which should now be confirmed in a larger cohort. Although further research should seek to determine which proteins are the most informative, we have demonstrated the value of combining PCMRI with CSF biomarker assays. Recent studies have emphasized how difficult it is to diagnose Alzheimer’s disease—especially in patients with normal pressure hydrocephalus (Graff-Radford, 2014). CSF biomarkers and proteomic methods (Fonteh et al., 2006) are of value for the diagnosis of neurodegenerative disorders (Davidsson et al., 2002; Seppälä et al., 2012; Sweeney et al., 2015) and neurovascular ischemic disorders (Siman et al., 2005; Vilar-Bergua et al., 2015). Combining these biological tools with PCMRI (and not just conventional MRI) may improve the diagnosis and monitoring of these conditions.

## Author Contributions

VP: Data analysis, writing and editing the manuscript. OB: project design, acquisition and post-processing of MRI data, data analysis, writing and editing the manuscript. JZ-A: clinical investigation. CC: neurosurgical procedures. RB: statistical and data analyses. J-MS: ethical approval, project design. A-MB: biochemical assays. JZ-A: project design.

## Conflict of Interest Statement

The authors declare that the research was conducted in the absence of any commercial or financial relationships that could be construed as a potential conflict of interest.

## Abbreviations

CC: cardiac cycle
CH: chronic hydrocephalus
CSF: cerebrospinal fluid
ICP: intracranial pressure
L/V: lumbar/ventricular
MRI: magnetic resonance imaging
PCMRI: phase-contrast magnetic resonance imaging
SV_aqu_: stroke volume measured at the cerebral aqueduct
SV_spine_: stroke volume measured at the spinal canal at C2-C3
CCT_aqu_: cardiac cycle time measured at the cerebral aqueduct
CCT_spine_: cardiac cycle time measured at the spinal canal at C2-C3
V_exp_: expected ventricular CSF protein level
V_m_: measured ventricular CSF protein level.
